# VV-YOLO: A Vehicle View Object Detection Model Based on Improved YOLOv4

**DOI:** 10.3390/s23073385

**Published:** 2023-03-23

**Authors:** Yinan Wang, Yingzhou Guan, Hanxu Liu, Lisheng Jin, Xinwei Li, Baicang Guo, Zhe Zhang

**Affiliations:** 1 China FAW Corporation Limited, Global R&D Center, Changchun 130013, China; 2 School of Vehicle and Energy, Yanshan University, Qinhuangdao 066000, China

**Keywords:** object detection, deep learning, vehicle view, YOLOv4, network optimization

## Abstract

Vehicle view object detection technology is the key to the environment perception modules of autonomous vehicles, which is crucial for driving safety. In view of the characteristics of complex scenes, such as dim light, occlusion, and long distance, an improved YOLOv4-based vehicle view object detection model, VV-YOLO, is proposed in this paper. The VV-YOLO model adopts the implementation mode based on anchor frames. In the anchor frame clustering, the improved K-means++ algorithm is used to reduce the possibility of instability in anchor frame clustering results caused by the random selection of a cluster center, so that the model can obtain a reasonable original anchor frame. Firstly, the CA-PAN network was designed by adding a coordinate attention mechanism, which was used in the neck network of the VV-YOLO model; the multidimensional modeling of image feature channel relationships was realized; and the extraction effect of complex image features was improved. Secondly, in order to ensure the sufficiency of model training, the loss function of the VV-YOLO model was reconstructed based on the focus function, which alleviated the problem of training imbalance caused by the unbalanced distribution of training data. Finally, the KITTI dataset was selected as the test set to conduct the index quantification experiment. The results showed that the precision and average precision of the VV-YOLO model were 90.68% and 80.01%, respectively, which were 6.88% and 3.44% higher than those of the YOLOv4 model, and the model’s calculation time on the same hardware platform did not increase significantly. In addition to testing on the KITTI dataset, we also selected the BDD100K dataset and typical complex traffic scene data collected in the field to conduct a visual comparison test of the results, and then the validity and robustness of the VV-YOLO model were verified.

## 1. Introduction

As a key technology that can effectively alleviate typical traffic problems and improve traffic safety, an intelligent transportation system has been fully developed around the world [1,2]. The large-scale application of autonomous driving technology has become an inevitable choice for the development of modern transportation [3]. Environmental awareness technology is the key to realizing autonomous driving and the basis for subsequent path planning and decision control of autonomous vehicles. As an important branch of environmental perception technology, object detection from the vehicle perspective is tasked with predicting the position, size, and category of objects in the area of interest in front of the vehicle [4], which directly affects the performance of the environmental perception system of autonomous vehicles.

In terms of sensors used for vehicle-mounted visual angle object detection, visual sensors have become the most used sensors for object detection due to their ability to obtain abundant traffic information, low cost, easy installation, and high stability [5,6,7]. With the continuous development of hardware systems, such as graphics cards and computing units, object detection based on deep learning is the mainstream of current research [8,9]. With its advantages of high robustness and good portability, object detection of four-wheeled vehicles, two-wheeled vehicles, and pedestrians has been realized in many scenes.

In the field of object detection, the deep learning-based object detection model can be divided into two stages, one of which stage is according to the implementation logic. The two-stage object detection model is usually composed of two parts: region of interest generation and candidate box regression. The R-CNN series [10,11,12,13] model, R-FCN [14], SPP [15], and other structures are the representatives of the two-stage object detection model. The two-stage object detection model has made a great breakthrough in precision performance, but it is difficult to use in embedded platforms with insufficient computing power, such as roadside units and domain controllers, which also promotes the birth of the single-stage object detection model. The single-stage object detection model treats the object detection task as a regression problem. By designing the network structure of the end-to-end mode, the feature extraction of the input image is carried out directly, and the prediction results are output. Early single-stage object detection models mainly include YOLO [16] and SSD [17]. Such models have great advantages in inference speed, but their detection precision is lower than that of the two-stage model. Due to this, the balance between detection precision and inference speed has become the focus of single-stage object detection model research and achieved rapid development in recent years. Excellent models, such as RetinaNet [18], YOLOv4 [19], CornerNet [20], and YOLOv7 [21], have emerged.

Table 1 shows the representative work in the field of vehicle-view object detection in recent years. Although these studies can solve the problem of object detection in complex vehicle-view scenes to a certain extent, they usually need to introduce additional large modules, such as the GAN [22] network and its variants, or just study a single object, such as pedestrians or vehicles. However, the autonomous vehicle needs to pay attention to three objects—a four-wheel vehicle, two-wheel vehicle and pedestrian—from the onboard perspective at the same time, and the computing power of its computing platform is limited, so the precision and real-time performance cannot be taken into account.

Inspired by the above research results and remaining problems, this paper proposes a vehicle view object detection model, VV-YOLO, based on improved YOLOv4. This model adopts the end-to-end design idea and optimizes the YOLOv4 benchmark model from three aspects: anchor frame clustering algorithm, loss function and neck network. Firstly, the improved K-means++ [28] algorithm is used to achieve more accurate and stable anchor frame clustering for the experimental dataset, which is a prerequisite for the target detection model based on the anchor frame to obtain a model with excellent performance. Secondly, a focal loss [18] loss function was introduced in the model training part to improve the feature extraction ability of the model for the target of interest in complex scenes. Finally, combined with the coordinate attention module [29], the CA-PAN neck network was proposed to model the channel relationship of image features, which could greatly improve the model’s attention at the region of interest.

## 2. Related Works

### 2.1. Structure of the YOLOv4 Model

**Figure 1:**
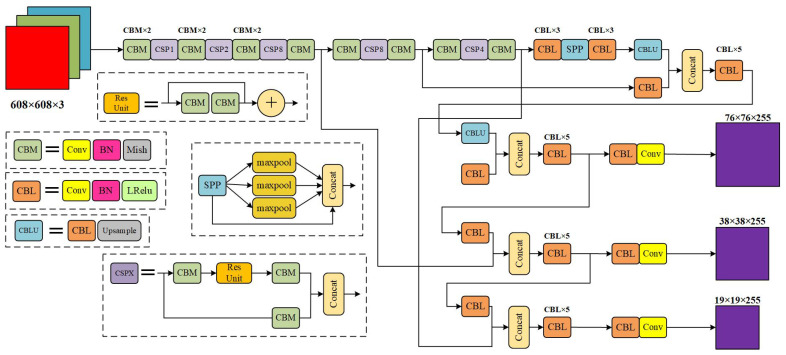
YOLOv4 model structure.

In 2020, Alexey Bochkovskiy et al. [30] improved YOLOv3 with a lot of clever optimization ideas and then proposed YOLOv4. Figure 1 shows its network structure. The design idea of YOLOv4 is consistent with that of YOLO. It is also a single-stage model, which can be divided into three parts: backbone network, neck network and detection network. The backbone network is called CSPDarkNet53 [19]. Different from the DarkNet53 [30] used in YOLOv3, it uses a cross-stage hierarchical structure for network connection, which reduces the amount of computation and ensures the feature extraction effect. The neck network of YOLOv4 was constructed using the PAN [31] path aggregation network, which improved the fusion effect of multilevel features compared to the FPN [32] feature pyramid network. In addition, YOLOv4 also uses the SPP network in front of the neck network to enrich the receptive field of image features. After the output features of the neck network are obtained, the input features are decoded by the prediction head of three scales to realize the perception of the large, medium and small-scale objects.

YOLOv4 still applies the strategy of prior box and batch standardization from YOLOv2 [33] to ensure the regularity of model training parameters. Meanwhile, the Mish [34] activation function was introduced in YOLOv4 to make the training gradient descent smoother. Compared with the ReLU [35] activation function, the possibility of loss falling into local minimization was reduced. In addition, YOLOv4 also used Mosaic [19] data enhancement and DropBlock [36] regularization to reduce the overfitting of the model.

### 2.2. Loss Function of the YOLOv4 Model

The loss function of the YOLOv4 is composed of regression loss, confidence loss and classification loss. Different from the function adopted by other YOLO models, YOLOv4 uses the CIoU [37] function to construct the model intersection ratio loss function. It uses the diagonal distance of the minimum enclosure box to formulate a penal strategy to further reduce the false detection rate of the small-scale objects. However, in the class loss function, the cross-entropy function is still adopted.

(1) $$\begin{array}{ll} & {L=\lambda }_{\mathrm{coord}\;}\sum_{i=0}^{K\times K}\sum_{j=0}^{M}I_{\mathrm{ij}}^{\mathrm{obj}\;}\left({2-w}_{i}{\times h}_{i}\right)(1-\mathrm{CIoU})-\\ & \;\sum_{i=0}^{K\times K}\sum_{j=0}^{M}I_{\mathrm{ij}}^{\mathrm{obj}\;}\left[{\hat{C}}_{i}\log\left(C_{i}\right)+\left(1-{\hat{C}}_{i}\right)\log\left({1-C}_{i}\right)\right]-\\ & \lambda_{\mathrm{noobj}\;}\sum_{i=0}^{K\times K}\sum_{j=0}^{M}I_{\mathrm{ij}}^{\mathrm{noobj}\;}\left[{\hat{C}}_{i}\log\left(C_{i}\right)+\left(1-{\hat{C}}_{i}\right)\log\left({1-C}_{i}\right)\right]-\\ & \;\sum_{i=0}^{K\times K}\sum_{i=0}^{M}I_{\mathrm{ij}}^{\mathrm{obj}\;}\sum_{c\in \mathrm{classes}}\left[{\hat{p}}_{i}(c)\log\left(p_{i}\left(c\right)\right)+\left(1-{\hat{p}}_{i}\left(c\right)\right)\log\left({1-p}_{i}\left(c\right)\right)\right] \end{array}$$

In Equation (1), K × K represents the mesh size, which can be 19 × 19, 38 × 38 or 76 × 76. M represents the detection dimension, whose value is 3.${\;\lambda }_{\mathrm{coord}}$ represents the positive sample weight coefficient, whose value is generally 1. The values of $I_{\mathrm{ij}}^{\mathrm{obj}}$ and $I_{\mathrm{ij}}^{\mathrm{noobj}}$ are either 0 or 1, which is used to judge the positivity or negativity of the sample. ${\hat{C}}_{i}$ and $C_{i}$ represent the sample and predicted values, respectively. $\left({2-w}_{i}{\;\times \;h}_{i}\right)$ is used to punish the smaller prediction box. $w_{i}$ and $h_{i}$ indicate the width and height of the center point of the prediction box, respectively. The CIoU’s equation is shown below.

(2) $$\mathrm{CIoU}=\mathrm{IoU}-\frac{\rho^{2}{(b,b}^{\mathrm{gt}})}{c^{2}}-\beta \nu$$

In Equation (2),$\;\rho^{2}{(b,b}^{\mathrm{gt}})$ represents the Euclidean distance between the center point of the prediction box and the real box, and c represents the diagonal distance between the minimum closure region that can contain both the prediction box and the real box. β is the parameter measuring the consistency of the aspect ratio, and ν is the tradeoff parameter. The calculation equations are shown in Equations (3) and (4), respectively.

(3) $$\beta =\frac{\nu }{1-\mathrm{IoU}+\nu }$$

(4) $$\nu =\frac{4}{\pi^{2}}{(\arctan\frac{w^{\mathrm{gt}}}{h}-\arctan\frac{w}{h})}^{2}$$

### 2.3. Discussion on YOLOv4 Model Detection Performance

**Figure 2:**
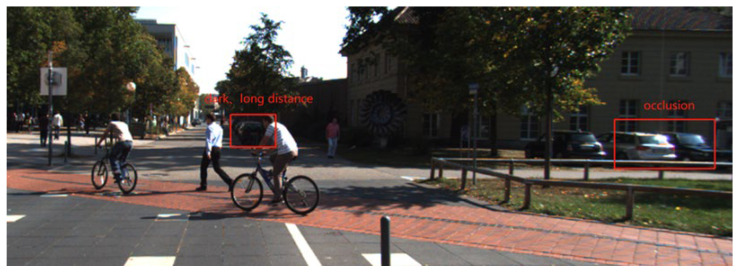
Typical scene from vehicle view.

As an advanced single-stage target detection model, YOLOv4 has a great advantage over the two-stage target detection model with detection speed. It can achieve a balance between precision and speed in conventional scenarios and meet the basic requirements of an automatic driving system. Figure 2 is a typical scene from the vehicle mount’s perspective. As can be seen from the figure, complex situations, such as dark light, occlusion and distance, are prone to occur under the vehicle-mounted perspective, and multiple types of traffic targets are often included. In the face of such scenarios, the YOLOv4 model’s ability to learn and extract effective features of the target is reduced, often resulting in missed detection and false detection. It can be seen that the current problem that urgently needs to be solved is object detection under the unfavorable conditions of the vehicle-view angle. Therefore, starting with the model structure and training strategy, this paper uses targeted design to improve the image feature modeling ability of the YOLOv4 model and improve the learning and extraction effects of effective features of the model in occlusion, dark light and other scenes, and proposes the vehicle-mounted perspective target detection model VV-YOLO.

## 3. Materials and Methods

### 3.1. Improvements to the Anchor Box Clustering Algorithm

**Figure 3:**
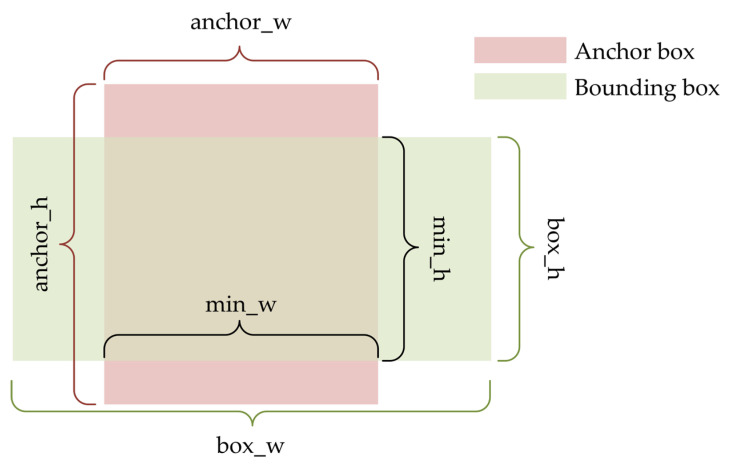
Illustration of the IoU calculation.

For the object detection model based on the regression anchor box, the size of the anchor box is usually set by the clustering algorithm, and the YOLOv4 model uses the K-means clustering algorithm [38]. First, randomly select all the original anchor boxes from all the real boxes, and then adjust the position of the anchor boxes by comparing the IoU of each original anchor box to the real box, and then get the new anchor frame size. Repeat the above steps until all the anchor boxes no longer change. According to the position relationship between the anchor box and the bounding box in Figure 3, the formula for calculating the IoU can be obtained, as shown in Equation (5).

(5) $$\mathrm{IoU}=\frac{|\mathrm{Anchor}\;\mathrm{box}\;\cap \;\mathrm{Bounding}\;\mathrm{box}|}{|\mathrm{Anchor}\;\mathrm{box}\;\cup \;\mathrm{Bounding}\;\mathrm{box}|}$$

**Figure 4:**
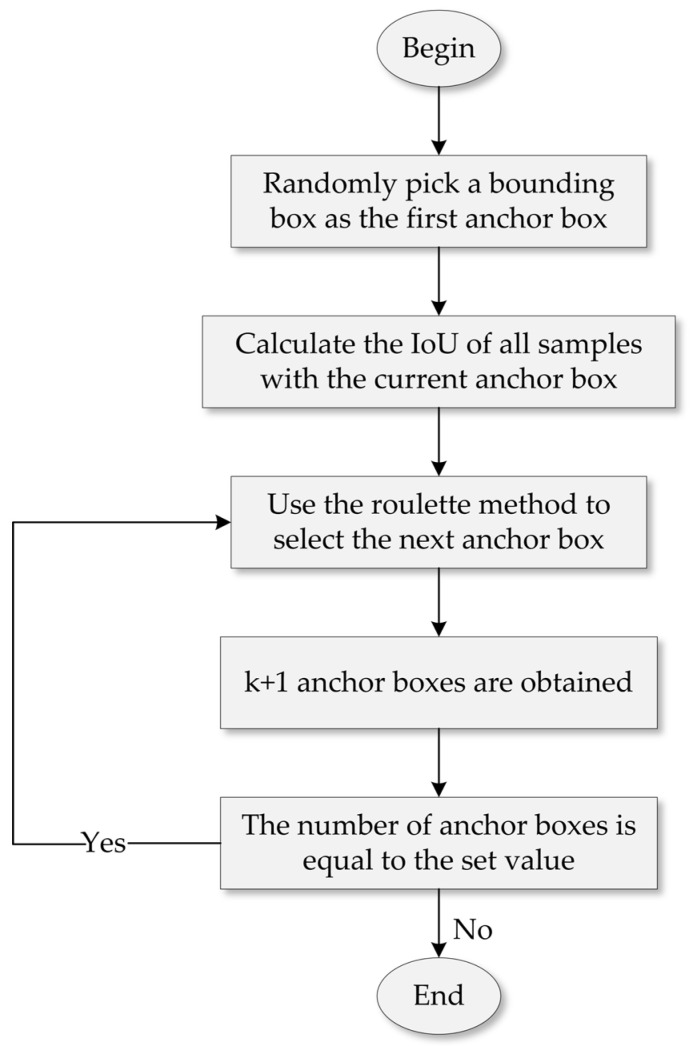
Improved K-means++ algorithm logic.

The clustering effect of the anchor frame of the YOLOv4 model depends on the random setting of the original anchor box, which has great uncertainty and cannot guarantee the clustering effect, and it usually takes multiple experiments to obtain the optimal anchor box size. In order to avoid the bias and instability caused by the random setting of points, the VV-YOLO model is based on the improved K-means++ clustering algorithm, which is used for the anchor box coordinate setting of experimental data, and its implementation logic is shown in Figure 4.

The essential difference between the improved K-means++ algorithm and the K-means algorithm is reflected in the initialization of the anchor box size and the method of the anchor frame selection. The former first randomly initializes a real box as the original anchor box, and secondly, each real box uses Equation (1) to calculate the difference value from the current anchor box, and the difference value calculation formula is shown in Equation (6).

(6) d(box,centroid)=1-IoU(box,centroid)

In Equation (6), *box* represents the current anchor box, *centroid* represents a sample of data; *IoU* represents the intersection and union ratio of the data sample to the current anchor box.

After the variance value is calculated, a new sample is selected as the next anchor frame using the roulette method until all anchor frames are selected. The principle of selection is that samples that differ significantly from the previous anchor box have a higher probability of being selected as the next anchor box. The following mathematical explanation is given for it:

Suppose the minimum difference value of N samples to the anchor box is $\left\{D_{1}{,D}_{2}{,D}_{3}{\ldots D}_{N}\right\}\;$, and then use Equation (7) to calculate the sum of the minimum differences from N samples to the current anchor box. Then, randomly select a value that does not exceed Sum, use Equation (8) to iteratively calculate the difference, stop calculating when r is less than 0, and the resulting point is the new anchor box size.

(7) $${\mathrm{Sum}=D}_{1}{+D}_{2}{+\ldots +D}_{N}$$

(8) $${r=r\;-\;D}_{N}$$

**Figure 5:**
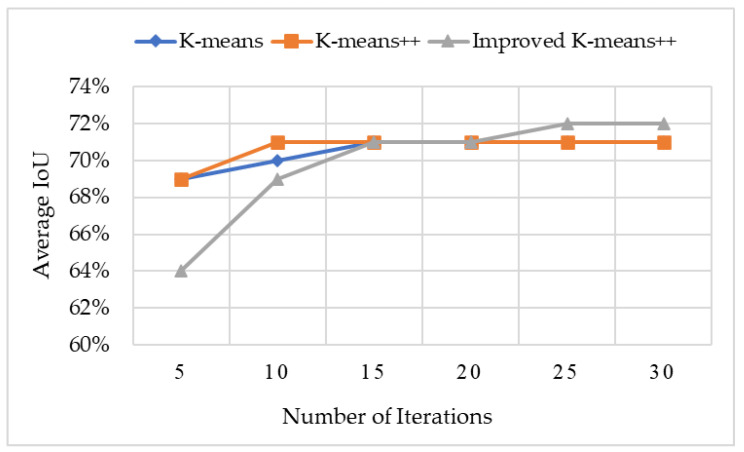
The clustering effect of two clustering algorithms on KITTI dataset.

**Figure 6:**
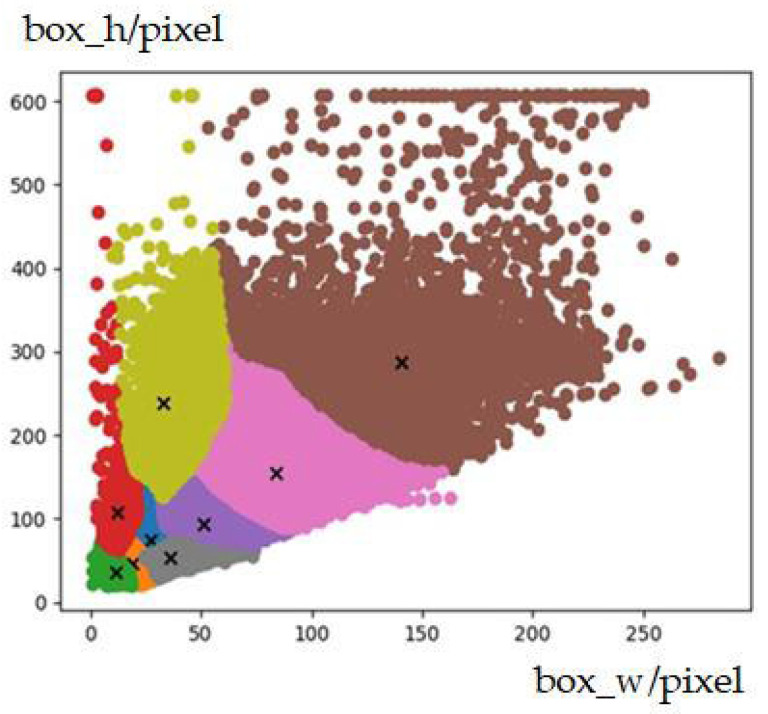
Cluster results of the improved K-means++ algorithm on the KITTI dataset.

Figure 5 shows the comparison of the average results of multiple clusters of K-means, K-means++ and improved K-means++ on the KITTI dataset [39]. The abscissa represents the number of iterations of the clustering algorithm, and the abscissa represents the average intersection ratio (IoU) of the obtained anchor box and all real boxes. Figure 6 shows the anchor box clustering results of the improved K-means++ algorithm. The results in the above figure show that the improved K-means++ algorithm can obtain a better clustering effect, and its average intersection union ratio is 72%, which is better than the K-means and K-means++ algorithms, which verifies its effectiveness.

### 3.2. Optimization of the Model Loss Function Based on Sample Balance

For the definition of samples in the YOLOv4 model, the concepts of the four samples are explained as follows:

1. The essence of object detection in the YOLOv4 model is to carry out intensive sampling, generate a large number of prior boxes in an image, and match the real box with some prior boxes. The prior box on the successful match is a positive sample, and the one that cannot be matched is a negative sample.
2. Suppose there is a dichotomous problem, and both Sample 1 and Sample 2 are in Category 1. In the prediction results of the model, the probability that Sample 1 belongs to Category 1 is 0.9, and the probability that Sample 2 belongs to Category 1 is 0.6; the former predicts more accurately and is an easy sample to classify; the latter predicts inaccurately and is a difficult sample to classify.

For deep learning models, sample balance is very important. A large number of negative samples will affect the model’s judgment of positive samples, and then affect the accuracy of the model, and the dataset will inevitably have an imbalance of positive and negative samples and difficult samples due to objective reasons. In order to alleviate the sample imbalance caused by the distribution of the dataset, this paper uses the focus function focal loss to reconstruct the loss function of the model and control the training weight of the sample.

From Equation (1) above, it can be seen that the confidence loss function of the YOLOv4 model is constructed using the cross-entropy function, which can be simplified to the following equation:

(9) $$L_{conf}=\sum_{i=0}^{K\times K}\sum_{j=0}^{M}I_{\mathrm{ij}}^{\mathrm{obj}\;}\left[-\log\left(C_{i}\right)\right]+\sum_{i=0}^{K\times K}\sum_{j=0}^{M}I_{\mathrm{ij}}^{\mathrm{noobj}\;}\left[-\log\left(C_{i}\right)\right]$$

The confidence function of YOLOv4 is reconstructed by using the focus function focal loss, and the loss function of the VV-YOLO model is obtained, as shown in Equation (10).

(10) $$\begin{array}{ll} & {L=\lambda }_{\mathrm{coord}\;}\sum_{i=0}^{K\times K}\sum_{j=0}^{M}I_{\mathrm{ij}}^{\mathrm{obj}\;}\left({2-w}_{i}{\;\times \;h}_{i}\right)(1-\mathrm{CIoU})-\\ & \;\sum_{i=0}^{K\times K}\sum_{j=0}^{M}I_{\mathrm{ij}}^{\mathrm{obj}\;}\left[\alpha_{t}{{(1-C}_{i})}^{\gamma }{\log(C}_{i})\right]-\\ & \lambda_{\mathrm{noobj}\;}\sum_{i=0}^{K\times K}\sum_{j=0}^{M}I_{\mathrm{ij}}^{\mathrm{noobj}\;}\left[\alpha_{t}{{(1-C}_{i})}^{\gamma }{\log(C}_{i})\right]-\\ & \;\sum_{i=0}^{K\times K}\sum_{i=0}^{M}I_{\mathrm{ij}}^{\mathrm{obj}\;}\sum_{c\in \mathrm{classes}}\left[{\hat{p}}_{i}(c)\log\left(p_{i}\left(c\right)\right)+\left(1-{\hat{p}}_{i}\left(c\right)\right)\log\left({1-p}_{i}\left(c\right)\right)\right] \end{array}$$

In Equation (10), $\alpha_{t}$ is the balance factor, which is used to balance the positive and negative sample weights; γ is the regulator, which is used to adjust the proportion of difficult and easy sample loss. In particular, when γ is 0, Equation (10) is the loss function of the YOLOv4 model.

In order to verify the validity of $\alpha_{t}$ and γ in the loss function of the VV-YOLO model, the following mathematical derivation is carried out in this section. To reduce the effect of negative samples, add a balance factor $\alpha_{t}$ to Equation (9), leaving aside the parameters that do not affect the result, to get Equation (11).

(11) $${\mathrm{CE}(C}_{i}{)=-\alpha }_{t}{\log(C}_{i})$$

In Equation (11), $\alpha_{t}$ ranges from 0 to 1, and $\alpha_{t}$ is α when the sample is positive, and $\alpha_{t}$ is 1-α when the sample is negative, as shown in Equation (12). It can be seen that by setting the value of α, it is possible to control the contribution of positive and negative samples to the loss function.

(12) $$\alpha_{t}=\{\begin{array}{l} \alpha \;\mathrm{if}\;\mathrm{sample}\;\mathrm{is}\;\mathrm{positive}\\ 1-\alpha \;\;\;\;\mathrm{otherwise} \end{array}$$

For verification of the effect of regulator γ, a part of Equation (10) can be taken and rewritten as the following equation:

(13) $$L_{\mathrm{fl}}=-{\hat{C}}_{i}{{(1\;-\;C}_{i})}^{\gamma }{\log(C}_{i})\;-\;\left(1\;-{\hat{\;C}}_{i}\right)C_{i}^{\gamma }\log\left({1\;-\;C}_{i}\right)$$

In the training of deep learning models, the gradient descent method is used to search for the optimal solution to the loss function. The gradient can indicate the training weight of different samples during the training process, and the gradient is related to the first-order partial derivative of the loss function, so using Equation (13) to find the first-order partial derivative of the variable $C_{i}$, we can obtain Equation (14).

(14) $$\frac{\partial L_{\mathrm{fl}}}{\partial C_{i}}={\hat{C}}_{i}{\gamma (1-C}_{i}{)}^{\gamma -1}{\log(C}_{i})\;-\;{\hat{C}}_{i}{{(1\;-\;C}_{i})}^{\gamma }\frac{1}{C_{i}}\;-\;\left(1\;-\;{\hat{C}}_{i}\right){\gamma C}_{i}{}^{{\hat{C}}_{i}-1}\log\left({1\;-\;C}_{i}\right)+\left(1\;-{\hat{\;C}}_{i}\right)C_{i}{}^{\gamma }\frac{1}{{1-C}_{i}}$$

Suppose that there are two sample points where ${\hat{C}}_{i}$ is 0 and the values of $C_{i}$ are 0.1 and 0.4, respectively. When γ is 0, that is, when the loss function is a cross-entropy function, the values of the partial derivative are 1.11 and 1.66, respectively; when γ is 2, the values of the partial derivative are 0.032 and 0.67, respectively. It can be seen that after setting a certain value for γ , the ratio of hard-to-distinguish samples to easy-to-distinguish samples is greatly increased, which increases the weight of difficult-to-distinguish samples in network training and effectively improves the problem of insufficient training caused by uneven data distribution.

### 3.3. Neck Network Design Based on Attention Mechanism

**Figure 7:**
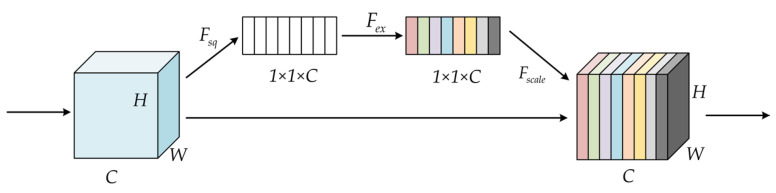
SENet model structure.

The attention mechanism in convolutional neural networks is a specific design that simulates the human brain, which can be introduced into multiple tasks in the field of computer vision and has the role of judging the importance of image features. The most classic attention mechanism network is SENet [40], whose structure is shown in Figure 7, which uses the global average pooling strategy and the fully connected layer to establish the interrelationship model between channels and effectively extract the importance of different channels.

**Figure 8:**
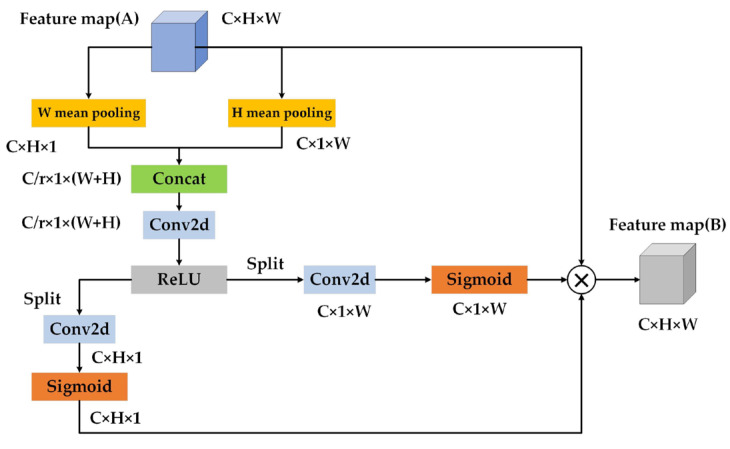
Coordinate attention module structure.

However, SENet only considers the importance of each channel by modeling channel relationships, ignoring the influence of feature location information on feature extraction. Considering the influence of the accuracy of feature position information on target detection accuracy, this paper chooses the coordinate attention network as a module introduced into the neck network; its structure is shown in Figure 8. In order to build an interaction model with accurate capture ability, each channel was coded along the horizontal and vertical coordinates, respectively. The coding formula is shown below.

(15) $$z_{c}=\frac{1}{H\times W}\sum_{i=1}^{H}\sum_{j=1}^{W}x_{c}\left(i,j\right)$$

(16) $$z_{c}^{h}\left(h\right)=\frac{1}{W}\sum_{0\leq i\leq W}x_{c}\left(h,i\right)$$

(17) $$z_{c}^{w}\left(w\right)=\frac{1}{H}\sum_{0\leq j\leq H}x_{c}\left(j,w\right)$$

In the above equation, x is the input. $z_{c}^{h}\left(h\right)$ and $z_{c}^{w}\left(w\right)$ are obtained by encoding each channel along the horizontal and vertical coordinates using a pooled kernel of size (H,1) or size (W,1) . This parallel modeling structure allows the attention module to capture one spatial direction while saving precise location information in another spatial direction, which helps the network more accurately mine out the object of interest. After the location information modeling is completed, the weights along the horizontal and vertical directions are obtained through the convolution operation and sigmoid function. The calculation formula for the output feature map is as follows:

(18) $$y_{c}\left(i,j\right){=x}_{c}{(i,j)\;\times \;g}_{c}^{h}{(i)\;\times \;g}_{c}^{w}\left(j\right)$$

According to the analysis of the YOLOv4 model in the previous article, based on the two existing improvement methods, a third improvement method is proposed to solve the problem of the declining feature extraction ability of the model. The coordinate attention module is introduced in the neck network of the YOLOv4 model, which improves the model’s attention to effective features by modeling the two dimensions of features and then improves the image feature extraction ability of the model.

**Figure 9:**
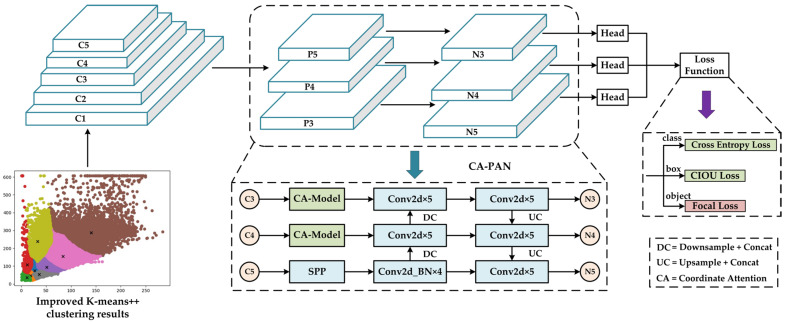
VV-YOLO model structure.

Considering that image features are transmitted differently in the backbone network and neck network, this paper hopes that the model can adaptively provide more training weight to effective features when the feature transfer mode changes, so as to reduce the impact of invalid features on the model’s training. Therefore, the coordinate attention module is inserted between the backbone network and the neck network, the CA-PAN neck network is designed and the VV-YOLO model shown in Figure 9 is finally formed.

## 4. Results and Discussion

### 4.1. Test Dataset

**Figure 10:**
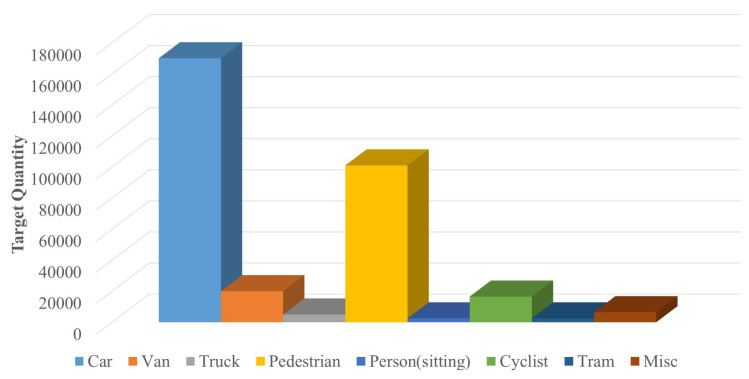
KITTI dataset data distribution.

The KITTI dataset [39], as the world’s largest computer vision algorithm evaluation dataset in unmanned driving scenarios, was jointly proposed by the Karlsruhe Institute of Technology in Germany and the Toyota Institute of Technology in the United States in 2012. The dataset can be used to evaluate multiple tasks in the computer vision field, including object detection, object tracking, visual odometry, etc. The data used to evaluate the object detection model in the KITTI dataset contains nearly 10,000 images in eight categories, including car, van, truck, pedestrian, person (sitting), cyclist, tram and misc, marking more than 200,000 objects in total. The data distribution is shown in Figure 10.

**Figure 11:**
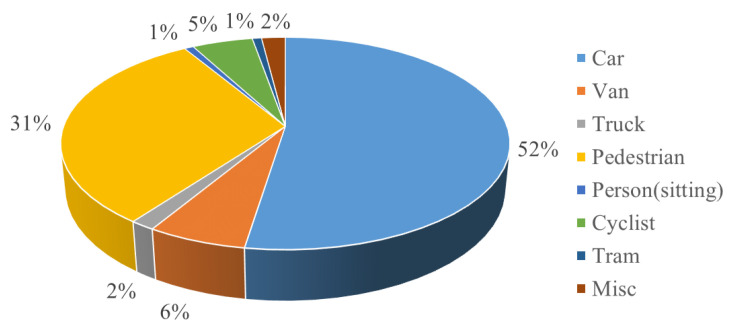
The proportion of various objects in the KITTI dataset.

Figure 11 shows the proportion of various objects in the object detection data. It can be found that the number of car classes far exceeds that of other categories, accounting for 52%, with serious sample imbalance. From the point of view of model hyperparameter tuning, highly unbalanced data distribution will seriously affect the fitting effect. According to the characteristics of traffic scene from a vehicle’s perspective and the objects of interest studied in this paper, a Python script is written to merge eight types of objects in the KITTI dataset into vehicle, pedestrian and cyclist [41]. The Vehicle class is composed of car, Van, truck, tram and misc. The Pedestrian class consists of pedestrian and person (sitting).

### 4.2. Index of Evaluation

In order to evaluate different object detection algorithms reasonably in an all-round way, it is usually necessary to quantify the performance of object detection algorithms from the real-time and precision perspectives. Reasonable evaluation has important guiding significance for selecting a reasonable object detection algorithm in different scenarios. For the object detection task from the vehicle view perspective, focus on precision, recall, average precision and real-time performance.

#### 4.2.1. Precision and Recall

In the field of machine learning, there are usually the following four relationship definitions for positive and negative sample relationships. TP (True Positive) is the correct positive sample, indicating that the negative sample is correctly identified. FP (False Positive) indicates the positive sample, indicating that the positive sample is incorrectly identified. FN (False Negative) is the wrong negative sample, indicating that the negative sample is identified incorrectly. TN (True Negative) indicates the correct negative sample, indicating that the negative sample is correctly identified.

**Figure 12:**
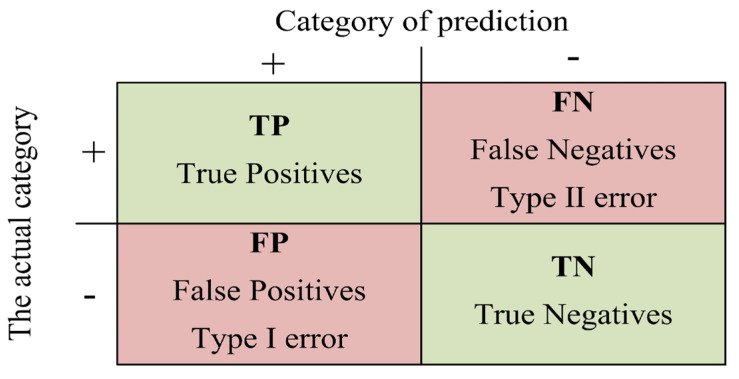
Confusion matrix structure.

The confusion matrix of the classical evaluation system of machine learning can be formed by arranging the above four positive and negative sample relations in matrix form, as shown in Figure 12.

According to the confusion matrix, the Precision and Recall of commonly used quantization methods can be defined. The precision represents the proportion of correct prediction of the model in all the results whose prediction result is a positive sample. The formula is shown in Equation (19).

(19) $$\mathrm{Precision}=\frac{\mathrm{TP}}{\mathrm{TP}+\mathrm{FP}}$$

Recall, also known as sensitivity, represents the proportion of correct model prediction among all the results whose true value is a positive sample, as shown in Equation (20).

(20) $$Recall=\frac{TP}{TP+FN}$$

#### 4.2.2. Average Precision

According to the above formula of precision and precision, it can be seen that the relationship between precision and precision is contradictory. If a single performance improvement is pursued, the performance of another index will often be sacrificed. Therefore, in order to comprehensively evaluate the object detection algorithm under different usage scenarios, PR curve is introduced.

The vertical coordinate of the PR curve is the precision under different confidence levels of detection boxes, and the horizontal coordinate is the precision under current confidence levels. The average precision is defined as the area under the PR curve, and its formula is shown in Equation (21).

(21) $$AP=\int_{0}^{1}PRdR$$

When evaluating the object detection model, the average precision of each type of object will be averaged to get mAP. mAP is one of the most commonly used evaluation means, and its size is between 0 and 1. Generally, the larger the mAP is, the better the performance of the object detection algorithm in terms of data. Its formula is shown in Equation (22).

(22) $$m\mathrm{AP}=\frac{1}{N}\sum_{i=1}^{N}{\mathrm{AP}}_{i}$$

### 4.3. VV-YOLO Model Training

Before model training, configuration files and super parameters need to be set. Configuration files mainly include category files and prior box files stored in txt file format. The category file stores the name of the object to be trained, and the prior box file stores the coordinates of the prior boxes of different sizes.

**Figure 13:**
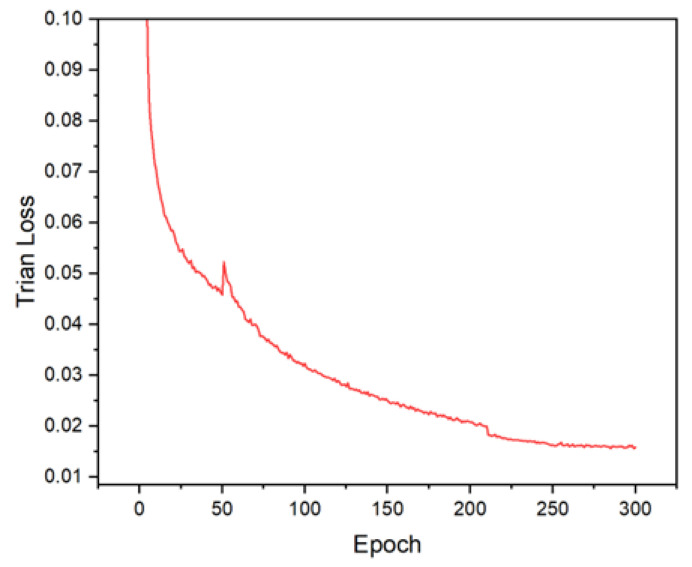
Training loss curve of VV-YOLO model.

**Figure 14:**
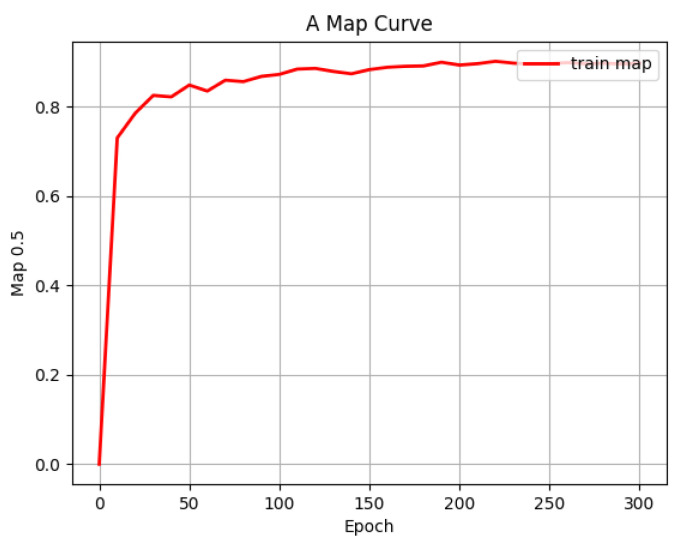
Training average precision change curve of VV-YOLO model.

The hyperparameters of the model training in this paper are set as follows:

- Input image size: 608 × 608;
- Number of iterations: 300;
- Initial learning rate: 0.001;
- Optimizer: Adam;

In order to avoid the problem of not obvious feature extraction due to too random weights during model training, the strategy of transfer learning was adopted during VV-YOLO model training, that is, the pre-training model provided by YOLOv4 developers was loaded during training, so as to obtain stable training effects. The change curves of loss function value and training accuracy during model training are shown in Figure 13 and Figure 14, respectively. The loss function value and training accuracy eventually converge to about 0.015 and 0.88, achieving the ideal training effect.

### 4.4. Discussion

#### 4.4.1. Discussion on Average Precision of VV-YOLO Model

**Figure 15:**
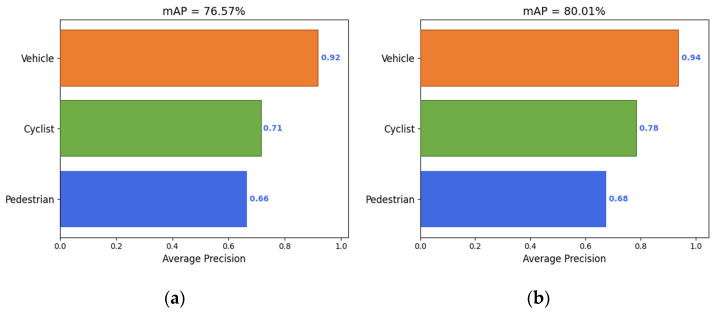
Schematic diagram of average precision: (**a**) YOLOv4; (**b**) VV-YOLO.

The YOLOv4 model and VV-YOLO model were used to test on the KITTI dataset [39], and the precision, recall and average precision results obtained were shown in the following table. According to the results in Table 2, the average precision of the VV-YOLO model is 80.01%, which is 3.44% higher than that of the YOLOv4 model. In terms of precision and recall, the VV-YOLO model is only lower than the YOLOv4 model in the recall of the pedestrian target, and the rest of the indicators have taken the lead. Figure 15 shows the average precision of the three types of objects of the two models, and the results show that the VV-YOLO model is superior to the YOLOv4 model.

To verify the effectiveness of each improved module of VV-YOLO, multiple rounds of ablation experiments were performed on the KITTI dataset, and the results are shown in the table below. From the results in the table, it can be concluded that the precision of the proposed model is improved by 6.88% and the average precision is improved by 3.44% with a slight increase in the number of parameters. Table 3 also shows the experimental results of comparison between the proposed model and a variety of advanced attention mechanisms, which also proves the effectiveness of the improved module.

In addition, six mainstream object detection models are selected for comparative testing, and Table 4 shows the precision, recall and average precision of the VV-YOLO model and the mainstream object detection model. From the results in the table, it can be concluded that the VV-YOLO model has achieved a leading position in other indicators except for slightly lower precision and recall than YOLOv5 and YOLOv4.

#### 4.4.2. Discussion on the Real-Time Performance of VV-YOLO Model

The weight size of VV-YOLO model is 245.73MB, only 1.29MB higher than that of the YOLOv4 model. On the NVIDIA GeForce RTX 3070 Laptop graphics card, the VV-YOLO model and seven mainstream object detection models were used to test and reason about the pictures in the KITTI dataset. Before the test and reasoning, the model would adjust the test pictures to the same pixel size.

After 100 inferences, the results of inference time and inference frames are shown in Table 5. The data transmission frame rate of the autonomous driving perception system is usually 15, and it is generally believed that the inference frame number of the object detection model is greater than 25 to meet the real-time requirements of the system, while the inference time of the VV-YOLO model is 37.19 ms, which is only 0.7 ms more than the YOLOv4 model, and the inference frame rate is 26.89. Compared with the YOLOv3 and YOLOv5 models, although the inference time of the VV-YOLO model has increased, its comprehensive performance is the best when combined with the precision test results.

#### 4.4.3. Visual Analysis of VV-YOLO Model Detection Results

**Figure 16:**
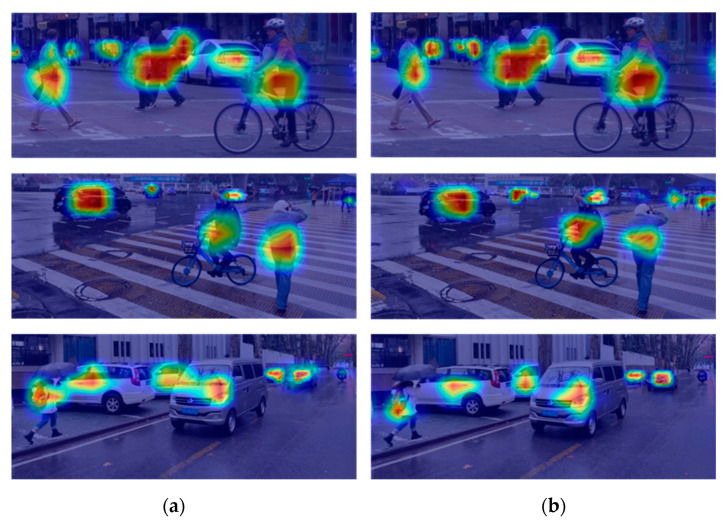
Object detection model inference heat map: (**a**) YOLOv4; (**b**)VV-YOLO.

**Figure 17:**
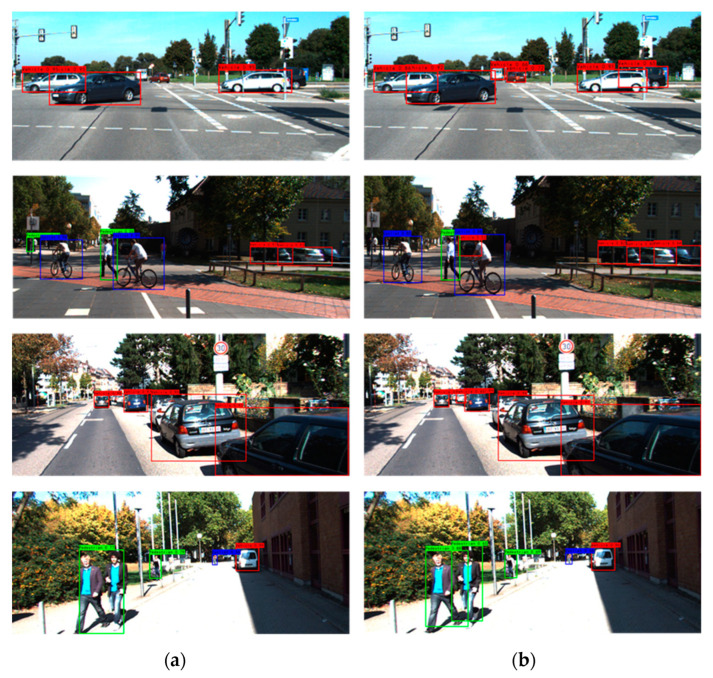
Object detection results of KITTI dataset: (**a**) YOLOv4; (**b**) VV-YOLO.

Figure 16 shows the model inference heat maps of the YOLOv4 model and the VV-YOLO model in multiple scenes from vehicle-mounted perspectives. The results in the figure show that, compared with YOLOv4, VV-YOLO can provide more attention to distant objects, occlusion and other objects. Figure 17 shows the detection results of YOLOv4 and VV-YOLO on the test data of the KITTI dataset. It can be seen that VV-YOLO can detect objects well when facing distant objects and occludes.

**Figure 18:**
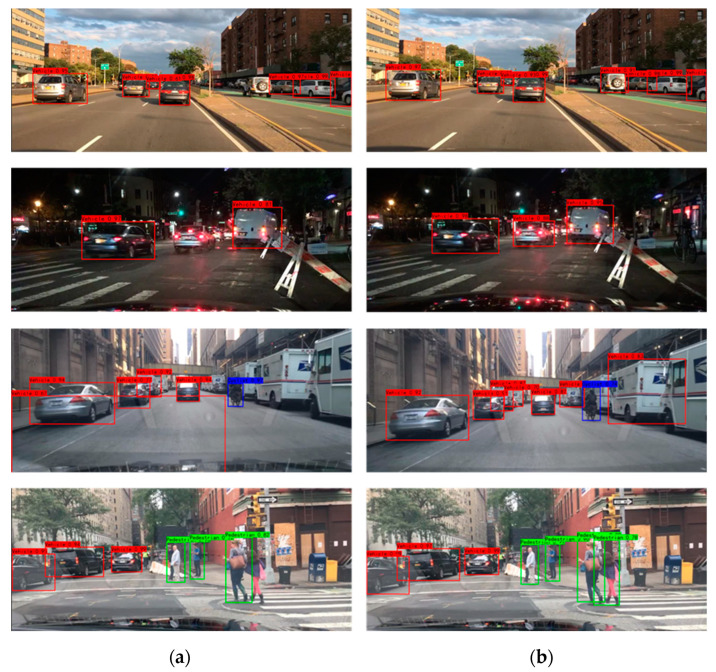
Object detection results of BDD100K dataset: (**a**) YOLOv4; (**b**) VV-YOLO.

**Figure 19:**
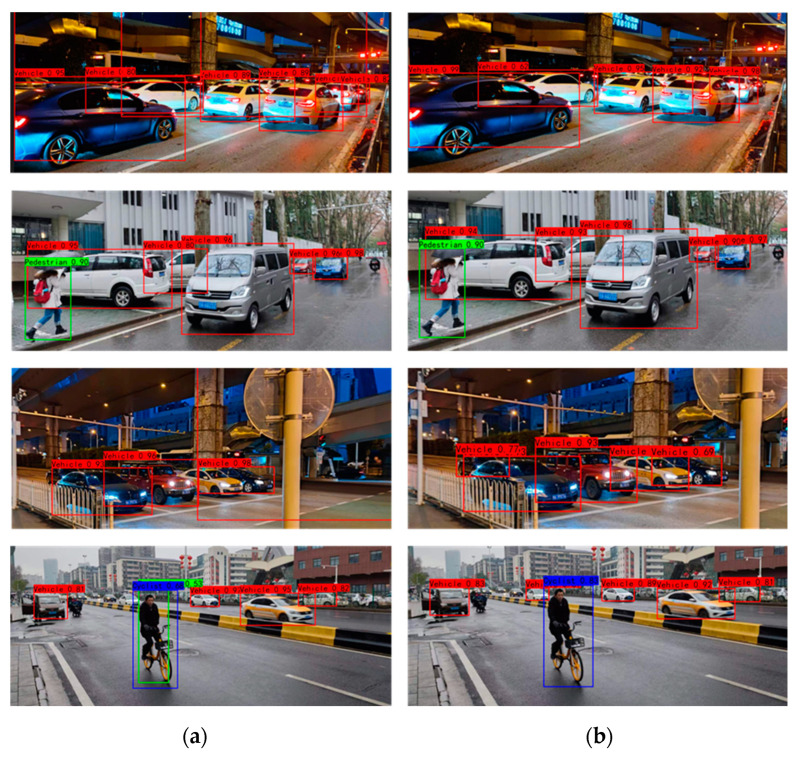
Object detection results of collected data: (**a**) YOLOv4; (**b**) VV-YOLO.

In order to verify the generalization performance of the VV-YOLO model, this paper also selected the BDD100K dataset and self-collected data in typical traffic scenes to conduct a comparison test of detection results. The test results are shown in Figure 18 and Figure 19. As can be seen from the results in the figure, the VV-YOLO model can detect both false detection and missing detection in the YOLOv4 model. The positive performance of the VV-YOLO model in actual scenarios is attributable to the specific design of the clustering algorithm, network structure and loss function in this paper.

## 5. Conclusions

Based on the end-to-end design idea, this paper proposes a vehicle viewing angle object detection model, VV-YOLO. Through the improved K-means++ clustering algorithm, fast and stable anchor box generation is realized on the model data side. In the VV-YOLO model training stage, the focus function focal loss is used to construct the model loss function, which alleviates the training imbalance caused by data distribution imbalance. At the same time, the coordinate attention mechanism is introduced into the model, and the CA-PAN neck network is designed to improve the learning ability of the model for the features of interest. In addition to the experiments on the experimental dataset, this study also collected some real road complex scene data in China for detection and comparison tests, and the visualization results confirmed the superiority of the VV-YOLO model. Several experimental results in this paper confirm that the improved model VV-YOLO can better realize object detection from the vehicle perspective and can take into account the precision and speed of model reasoning at the same time, which provides a new implementation idea for the autonomous vehicle perception module that has good theoretical and engineering practical significance.

## Figures and Tables

**Table 1:** Summary of literature survey on vehicle view object detection model.

Year	Title	Method	Limitation	Reference
2021	Vehicle Detection and Tracking in Adverse Weather Using a Deep Learning Framework	A visual enhancement mechanism was proposed and introduced into the YOLOv3 model to realize vehicle detection in snowy, foggy, and other scenarios.	There is the introduction of larger modules, and only the vehicle objects are considered.	[23]
2021	GAN-Based Day-to-Night Image Style Transfer for Nighttime Vehicle Detection	AugGAN network was proposed to enhance vehicle targets in dark light images, and the data generated by this strategy was used to train R-CNN and YOLO faster, which improved the performance of the object detection model under dark light conditions.	GAN networks are introduced, and multiple models need to be trained, and only vehicle objects are considered.	[24]
2022	SA-YOLOv3: An Efficient and Accurate Object Detector Using Self-Attention Mechanism for Autonomous Driving	A SA-YOLOv3 model is proposed, in which dilated convolution and self-attention module (SAM) are introduced into YOLOv3, and the GIOU loss function is introduced during training.	There are fewer test scenarios to validate the model.	[25]
2022	Feature Calibration Network for Occluded Pedestrian Detection	The fusion module of SA and FC features is designed, and FC-NET is further proposed to realize pedestrian detection in occlusion scenes	Only pedestrian targets are considered, and there are few verification scenarios.	[26]
2023	R-YOLO: A Robust Object Detector in Adverse Weather	QTNet and FCNet adaptive networks were proposed to learn the image features without tags and applied to YOLOv3, YOLOv5 and YOLOX, which improved the precision of object detection in foggy scenarios.	With the introduction of additional large networks, multiple models need to be trained.	[27]

**Table 2:** Test results of the YOLOv4 model and the VV-YOLO model on the KITTI dataset.

Evaluation Indicators		YOLOv4	VV-YOLO
**Precision**	Vehicle	95.01%	**96.87%**
	Cyclist	81.97%	**93.41%**
	Pedestrian	74.43%	**81.75%**
**Recall**	Vehicle	80.79%	**82.21%**
	Cyclist	55.87%	**55.75%**
	Pedestrian	56.58%	**52.24%**
**Average precision**		76.57%	**80.01%**

**Table 3:** Ablation experimental results of VV-YOLO model on the KITTI dataset.

Test Model		Precision	Recall	Average Precision
Baseline		83.80%	64.41%	76.57%
+Improved K-means++		89.83%	60.70%	77.49%
+Focal Loss		90.24%	61.79%	78.79%
attention mechanisms	+SENet	89.47%	62.99%	78.61%
	+CBAM	89.83%	60.69%	78.49%
	+ECA	89.66%	61.96%	78.48%
**VV-YOLO**		**90.68%**	**63.40%**	**80.01%**

**Table 4:** Comparative test results of the VV-YOLO model and mainstream object detection model.

Test Model	Precision	Recall	Average Precision
RetinaNet	90.43%	37.52%	66.38%
CenterNet	87.79%	34.01%	60.60%
YOLOv5	89.71%	61.08%	78.73%
Faster-RCNN	59.04%	76.54%	75.09%
SSD	77.59%	26.13%	37.99%
YOLOv3	77.75%	32.07%	47.26%
**VV-YOLO**	**90.68%**	**63.40%**	**80.01%**

**Table 5:** Real-time comparison between the VV-YOLO model and the mainstream object detection model.

Test Model	Inference Time	Inference Frames
RetinaNet	31.57 ms	31.67
YOLOv4	36.53 ms	27.37
CenterNet	16.49 ms	60.64
YOLOv5	26.65 ms	37.52
Faster-RCNN	62.47 ms	16.01
SSD	52.13 ms	19.18
YOLOv3	27.32 ms	36.60
**VV-YOLO**	**37.19 ms**	**26.89**

## Data Availability

Data and models are available from the corresponding author upon request.
